# Integrative ensemble learning framework for forecasting controlled drug release based on Raman spectral signatures

**DOI:** 10.1038/s41598-026-41837-0

**Published:** 2026-02-28

**Authors:** Ahmed H. Albariqi, Awaji Y. Safhi, Saad S. Alqahtani, Fahad Y. Sabei, Mahboubeh Pishnamazi

**Affiliations:** 1 Department of Pharmaceutics, College of Pharmacy, Jazan University, Jazan, 45142 Saudi Arabia; 2 Clinical Pharmacy Department, College of pharmacy, King Khalid University, Abha, Saudi Arabia; 3 Institute of Research and Development, Duy Tan University, Da Nang, Vietnam; 4 School of Engineering and Technology, Duy Tan University, Da Nang, Vietnam

**Keywords:** Controlled drug delivery, Diffusion-controlled kinetics, Polymer–drug interactions, Polysaccharide-based drug release, Process analytical technology, Statistical validation, Chemistry, Materials science, Mathematics and computing

## Abstract

Modeling drug-release kinetics from polysaccharide-coated oral controlled-release formulations remains challenging due to nonlinear diffusion–dissolution behavior, complex polymer–drug interactions, and the limited interpretability of conventional machine-learning approaches. In this study, we develop and validate a predictive framework for targeted colonic delivery of 5-aminosalicylic acid (5-ASA) from polysaccharide-coated solid oral dosage forms using Raman spectroscopy–derived molecular fingerprints and time-resolved dissolution data. The dataset comprises 155 formulation samples, each characterized by more than 1,500 Raman spectral features, categorical formulation variables (polysaccharide type and release medium), and drug-release measurements at 2, 8, and 24 h collected under simulated physiological conditions. A dual-optimizer, dual-ensemble learning strategy is introduced, integrating the Puma Optimizer Algorithm (POA) and Black-Winged Kite Algorithm (BWKA) within a Damsphere Weighted Ensemble (DWE) of XGBoost regression and AdaBoost models. The complementary exploration–exploitation dynamics of the two optimizers enhance convergence stability and generalization, yielding strong predictive performance under five-fold cross-validation (RMSE = 0.038; R^2^ = 0.991). Feature-level analysis based on F-statistics highlights release time, dissolution medium, and chemically meaningful Raman bands as dominant predictors, consistent with diffusion- and erosion-controlled release mechanisms in polysaccharide-coated systems. From a pharmaceutical perspective, the proposed framework reduces experimental burden while maintaining mechanistic interpretability, supporting Quality by Design (QbD) and green pharmaceutics principles. Owing to its modular architecture, the approach is readily extensible to other polymer-based oral controlled-release formulations and spectroscopic modalities.

**Supplementary Information:**

The online version contains supplementary material available at 10.1038/s41598-026-41837-0.

## Introduction

Controlled drug release has emerged as one of the key aspects of modern pharmaceutical research, aiming at the maintenance of therapeutic concentrations of the active agent for a longer period with a minimum of adverse effects and frequency of administration. This, therefore, requires an accurate release kinetics prediction in designing formulations to ensure reproducible bioavailability and patient compliance^1,2^. Apart from the therapeutic advantages, predictive modeling of drug release can support rational formulation design, reduce experimental workload, and accelerate regulatory approval by allowing in silico assessment of dissolution performance before physical trials. Predictive modeling of drug release has thus grown to become scientifically as well as industrially necessary with increasing demands for cost-effective and sustainable drug development pipelines from global healthcare systems^3,4^.

Traditional methods for understanding drug release have mainly depended on empirical and mechanistic models, such as the Higuchi, Korsmeyer-Peppas, and Hixson-Crowell equations, which describe diffusion and erosion-controlled kinetics through simplifying assumptions^5^. While these models have been instrumental in providing basic insights, they are fundamentally limited in their ability to capture nonlinear and multivariate interactions between formulation and environmental factors^6^. Similarly, the DoE method, although statistically rigorous, becomes computationally and experimentally heavy when multiple formulation variables interact in complex and non-additive ways^7^. These traditional methods frequently fail to account for high-dimensional data, such as that from Raman spectra, which capture complex physicochemical signals related to release dynamics^8^. Therefore, there remains a considerable gap between theoretical kinetic modeling and the predictive complexity needed for cutting-edge formulation design^9,10^.

The present study cannot be viewed independently but needs a full survey of the most recent studies controlling drug delivery and release prediction to know the substantial advancements made in the last two years in the field of drug delivery system materials, in the development of responsive drug release mechanisms, and the adoption of machine learning for the modeling of complex dissolution kinetics. Research has gone deep into polymeric matrices, hydrogels, composite capsules, technological evolutions like 3D printing, antibubbles, and microbiome-activated systems, all of which are contributing in different ways to the ultimate goal of release precision with improved biocompatibility^11–13^. In parallel, the adoption of data-driven models with hybrid algorithms has begun an unprecedented transformation in drug release predictive analytics, which enables the speedier optimization of formulations and identification of mechanisms. To establish the scientific basis for this study, Table 1 summarizes some key contributions from recent literature, covering works on experimental innovation in polymeric and hydrogel-based delivery systems to computation-based frameworks using machine learning for dissolution modeling^14,15^. Synthesis thus brings into light the diversity of the contemporary approaches and underlines the research gap that this current study tries to address with the development of a hybrid predictive framework that integrates ensemble learning with metaheuristic optimization to improve accuracy and interpretability in drug release modeling.

**Table 1:** Comparative summary of recent research on drug release prediction and formulation modeling.

Refs.	Main contribution and relevance
Abdalla et al.^16^	Developed machine-learning models using Raman spectra of polysaccharides to predict 5-ASA release from coated formulations. Demonstrated generalizability across simulated human, rat, and dog colonic environments and identified chemically relevant spectral features for rapid pre-screening of coating materials.
Lü et al.^17^	Reviewed polymer-based delivery systems for controlled release, highlighting diffusion-, erosion-, and swelling-controlled mechanisms and design principles linking polymer structure to release kinetics.
Kapoor et al.^18^	Reported pectin-based hydrogel formulations enabling controlled release via crosslinking density and pH sensitivity. Discussed formulation strategies and translational challenges related to stability and regulatory approval.
Yu et al.^19^	Investigated composite capsule systems combining natural and synthetic polymers to achieve dual- and multi-responsive release. Demonstrated improved encapsulation efficiency and spatially controlled release while noting scalability and biocompatibility limitations.
AL‑Rajabi et al.^20^	Proposed a hybrid machine-learning framework to predict release profiles and kinetic parameters of thermoresponsive hydrogels, improving formulation optimization and mechanistic interpretation compared with empirical fitting.
Kamath et al.^21^	Reviewed microbially activated drug delivery systems (MADDS), emphasizing enzyme- and metabolite-responsive release strategies for site-specific delivery and identifying microbiome variability as a key translational challenge.
Zia et al.^22^	Introduced particle-stabilized antibubbles as oral carriers enabling stimulus-triggered release under intestinal conditions and prolonged multi-site delivery in simulated gastrointestinal media.
Altalib^23^	Described 3D printing approaches for fabricating colon-targeted dosage forms with programmable geometry and release profiles, highlighting patient-specific dosing and regulatory considerations.
Subash et al.^24^	Reviewed lyotropic liquid crystalline nanoparticles for sustained release, showing phase-dependent release behavior and discussing challenges in scale-up and long-term stability.
Drapińska et al.^25^	Systematically reviewed sustained-release oral formulations of NSAIDs and paracetamol, identifying polymeric matrices and advanced manufacturing as key strategies while noting gaps between in vitro release and clinical outcomes.

**Fig. 1:**
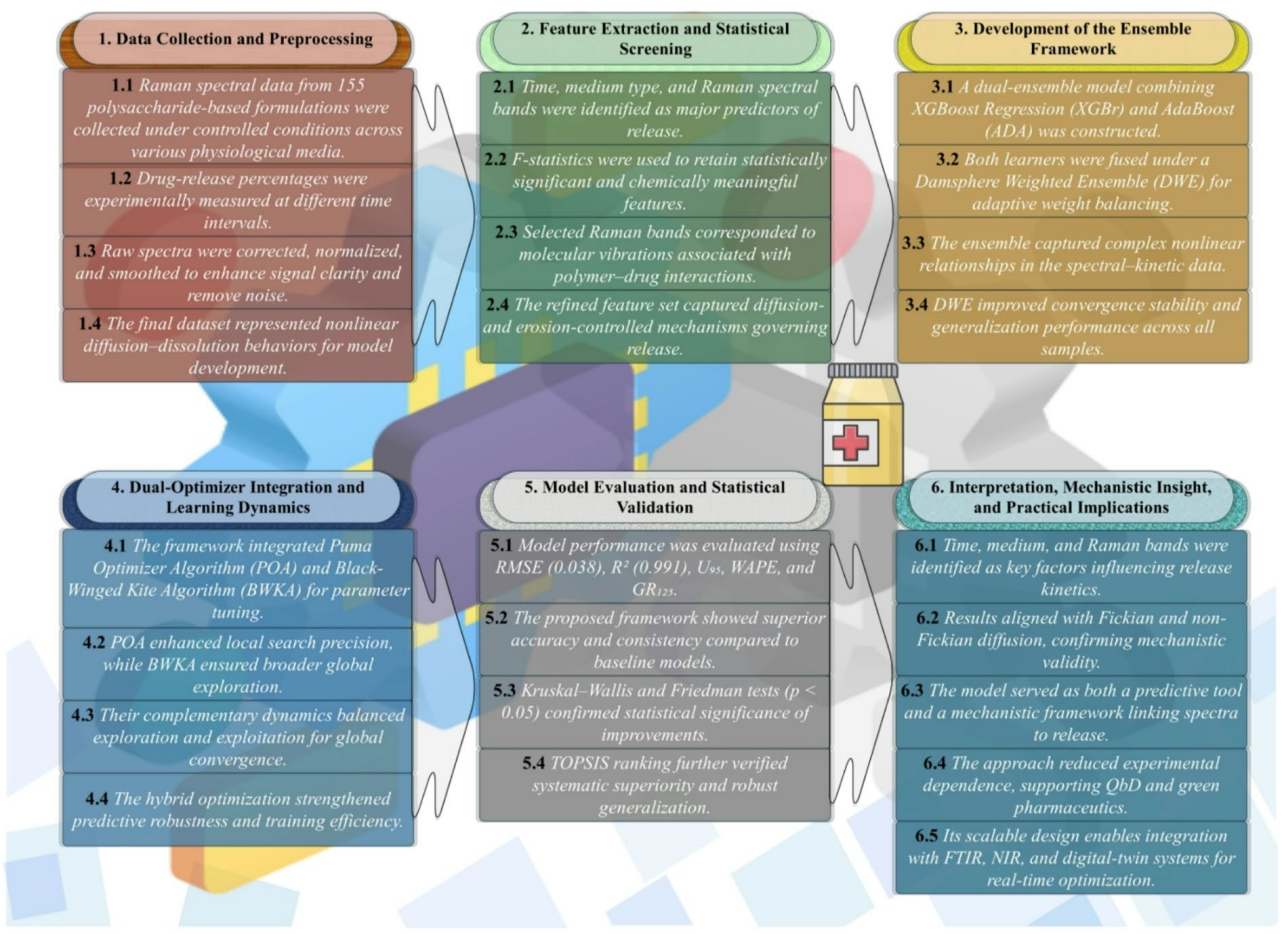
A schematic overview of the proposed dual-ensemble-dual-optimizer framework showing the sequential workflow.

Although recent advances in machine learning have improved the prediction accuracy of drug release profiles, most reported approaches rely on single learners or statically weighted ensembles with limited hyperparameter optimization and minimal mechanistic interpretation. As a result, these models often exhibit reduced robustness across physiological media and provide limited insight into how formulation attributes and molecular-level features govern release behavior. Moreover, the majority of existing studies treat prediction as the primary objective, with less emphasis on sensitivity analysis, feature relevance, or alignment with drug transport mechanisms. To address this gap, the present study proposes a dual-optimizer, dual-ensemble learning framework that integrates XGBoost Regression and AdaBoost within a Damsphere Weighted Ensemble, dynamically optimized using the Puma Optimizer Algorithm and the Black-Winged Kite Algorithm. Unlike conventional ensemble strategies with static configurations, the proposed framework embeds adaptive metaheuristic optimization to jointly refine model hyperparameters and ensemble weights, thereby enhancing convergence stability, generalization, and resistance to overfitting in high-dimensional Raman spectral spaces. Importantly, the framework is designed not only to improve predictive performance but also to elucidate the physicochemical drivers of drug release through sensitivity analysis and feature-level interpretation, thus strengthening the connection between data-driven modeling and polymer–drug transport theory. The comprehensive methodological workflow of the proposed predictive framework, summarizing each stage from data acquisition to mechanistic interpretation, is depicted in Fig. 1.

## Dataset and pre-processing methods

### Dataset description

While data-driven methodologies for drug release prediction have been increasingly developed, the models that have been created so far still lack sufficient hyperparameter optimization, suffer from inconsistent ensemble weighting, and are not easily adaptable to different physiological environments. These limitations have resulted in the inaccurate and non-generalizable modeling of nonlinear release kinetics originating from complex spectral and compositional data^26^. This study proposed a dual-optimizer ensemble framework combining XGBr and ADA models into a DWE, which is being optimized by both the POA and the BWKA. Such a hybridization broadens the exploration-exploitation balance during parameter tuning, thus enabling stable convergence and enhanced predictive synergy between the base learners. This work argues that, instead of using traditional ensemble techniques that depend on static parameter selection, one could embed adaptive metaheuristics to continually adjust model weights and hyperparameters, thereby making the model more resistant to overfitting and dataset variability. K-fold cross-validation is implemented to verify the stability of the proposed framework. Model performance is analyzed by means of various criteria, such as TOPSIS ranking, while non-parametric tests (Kruskal-Wallis and Friedman) are used to statistically confirm the results. Ultimately, the study goal is to present a computationally efficient, interpretable, and generalizable predictive model capable of drug release simulation from polysaccharide-based formulations and physiological media of different types.

### Data variables and feature characteristics

Each sample describes a multimodal variable set, which combines spectral, numerical, and categorical descriptors. More than 1,500 Raman spectral features constitute the heart of the dataset. These features represent the molecular vibrations, functional group interactions, and structural properties of the coating materials. Raman spectra are detailed physicochemical fingerprints that reflect changes in polymer morphology and composition, which are the main factors for drug release rates. Besides the numerical feature time, which indicates the duration of each release experiment, and two categorical features, polysaccharide name and medium, which provide the chemical and environmental context, are also well. These variables enable multiscale learning, where both molecular-level spectral patterns and macro-level experimental conditions are combined to obtain an accurate prediction of release kinetics. Moreover, the use of categorical and numerical context variables is crucial if the models are to capture cross-environment dependencies, which are necessary for generalizable drug-release prediction.

### Validation and statistical testing

In order to be rigorous in terms of methodology and have a strong generalization, the refined dataset was evaluated through a five-fold cross-validation (k-fold) design. Each fold had a balanced representation of the different types of polysaccharides and the media conditions. This technique restricts the overfitting issue and provides a precise measurement of the model’s performance in different settings. Additionally, a multi-criteria ranking of the model results has been done by TOPSIS with respect to all the implemented evaluation metrics, which ensures an unbiased comparison. Non-parametric tests, Kruskal-Wallis and Friedman, were performed across folds and models to ascertain statistical significance and result stability. These tests confirm that the observed differences in the model’s performance are due to the models being different rather than random chance, thereby increasing the reliability, reproducibility, and scientific integrity of the proposed predictive framework.

Table 2 provides a summary of the descriptive statistics of the input variables used in predictive modeling of drug release. The dataset is made up of the coating polysaccharides of the drug, which include Maize maltodextrin, Inulin (Orafti Synergy 1), Xylan, and Goji berry extract that have a wide range of numerical values, reflecting their physicochemical and structural heterogeneity. Average spectral intensities vary roughly from 1,099 to 370,000, which points to a significant molecular diversity for the coatings. The high standard deviations are evidence of the compositional variability, while most of the negative kurtosis values indicate that the stable, non-outlier distributions are suitable for robust machine learning. In contrast, positive kurtosis in Cook-up maize starch and Isomaltulose suggests a higher degree of uniformity in the structural composition. Temporal observations (2–24 h) and normalized release values (0-1.02) are sufficient to describe the dynamic dissolution behaviors across media and thus establish a reliable foundation for ensemble-based learning.

**Table 2:** Descriptive statistics of the input variables used for model development, computed directly from the underlying raw dataset provided as Supplementary Material. Reported values include minimum, maximum, mean, standard deviation, and kurtosis for each variable.

Distribution	Variable	Indicator Range				
Uniform	Medium	(1–4)				
		Min.	Max.	Avg.	St. Dev.	Kurtosis
Normal	Polysaccharide name	100.8	1987.1	1099.3	547.2	-1.18
	Maize maltodextrin	7357.1	54998.4	20438.0	11102.6	-0.64
	Inulin (Orafti Synergy 1)	7900.3	48575.5	21831.9	9629.6	-0.61
	Maltitol	7831.5	65478.5	21699.3	12994.6	0.31
	Inulin (Orafti HSI)	8120.8	54067.3	25161.1	12293.5	-0.96
	Pregelatinized starch (Starch 1500)	7430.8	66034.3	20586.7	11235.1	0.59
	Xylan	16240.2	94237.0	45776.0	20086.0	-0.64
	Resistant maize starch	15392.2	84803.6	34487.4	12408.5	0.46
	Goji berry extract	157191.3	571821.1	370559.9	114426.8	-0.94
	Coix lacryma esculentus extract	39648.3	134653.4	86764.1	25126.3	-0.93
	Cook up maize starch	6298.8	75355.0	20667.3	12744.9	1.28
	Raffinose	6027.9	23797.7	10855.0	3728.8	1.25
	Inulin (Orafti HP)	9105.8	51772.3	21770.8	9434.5	0
	Isomaltulose	7624.5	69537.0	19358.1	11440.2	3.30
	Time	2	24	11.35	9.31	-1.52
	Release	0	1.02	0.22	0.28	0.48

**Fig. 2:**
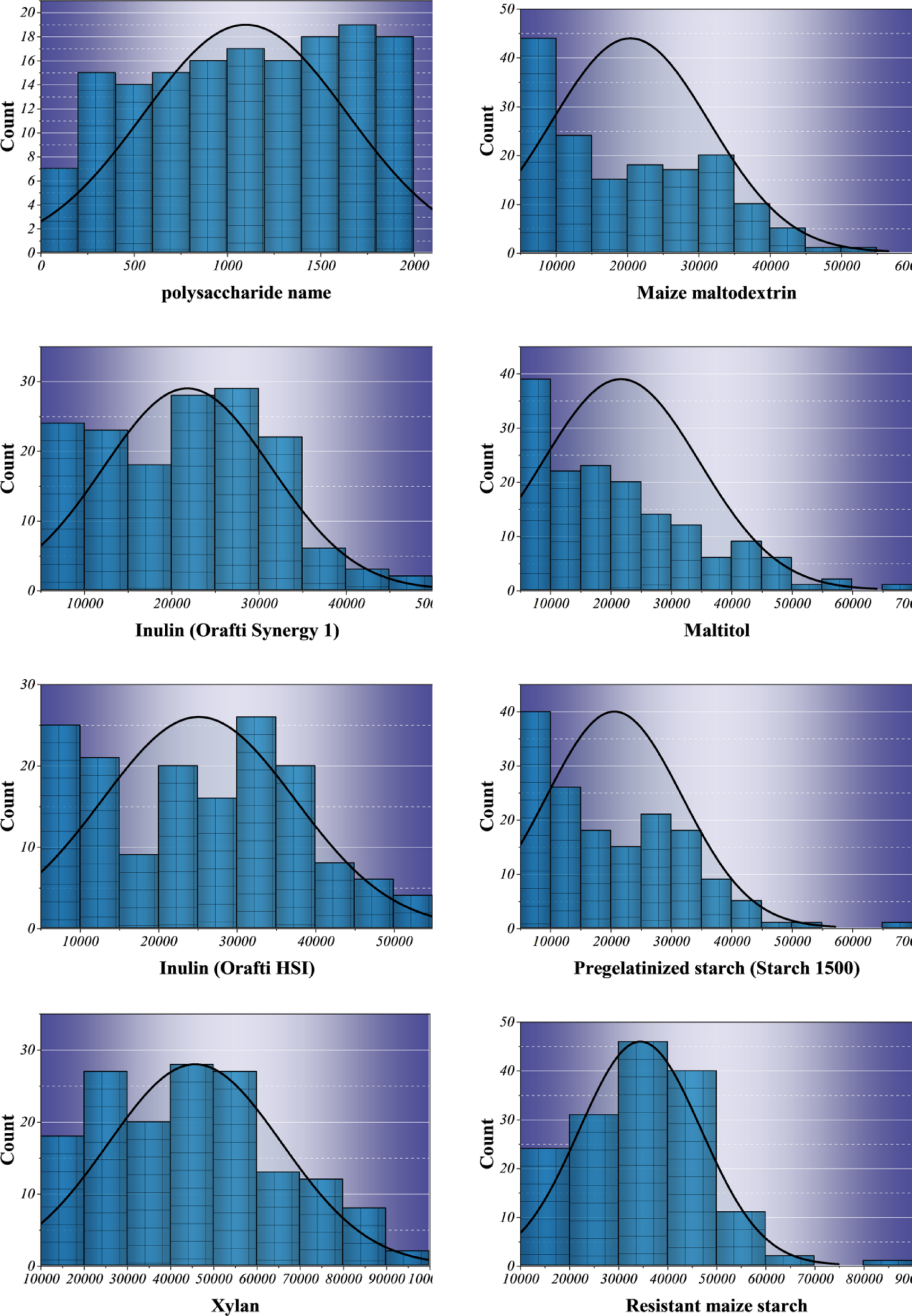

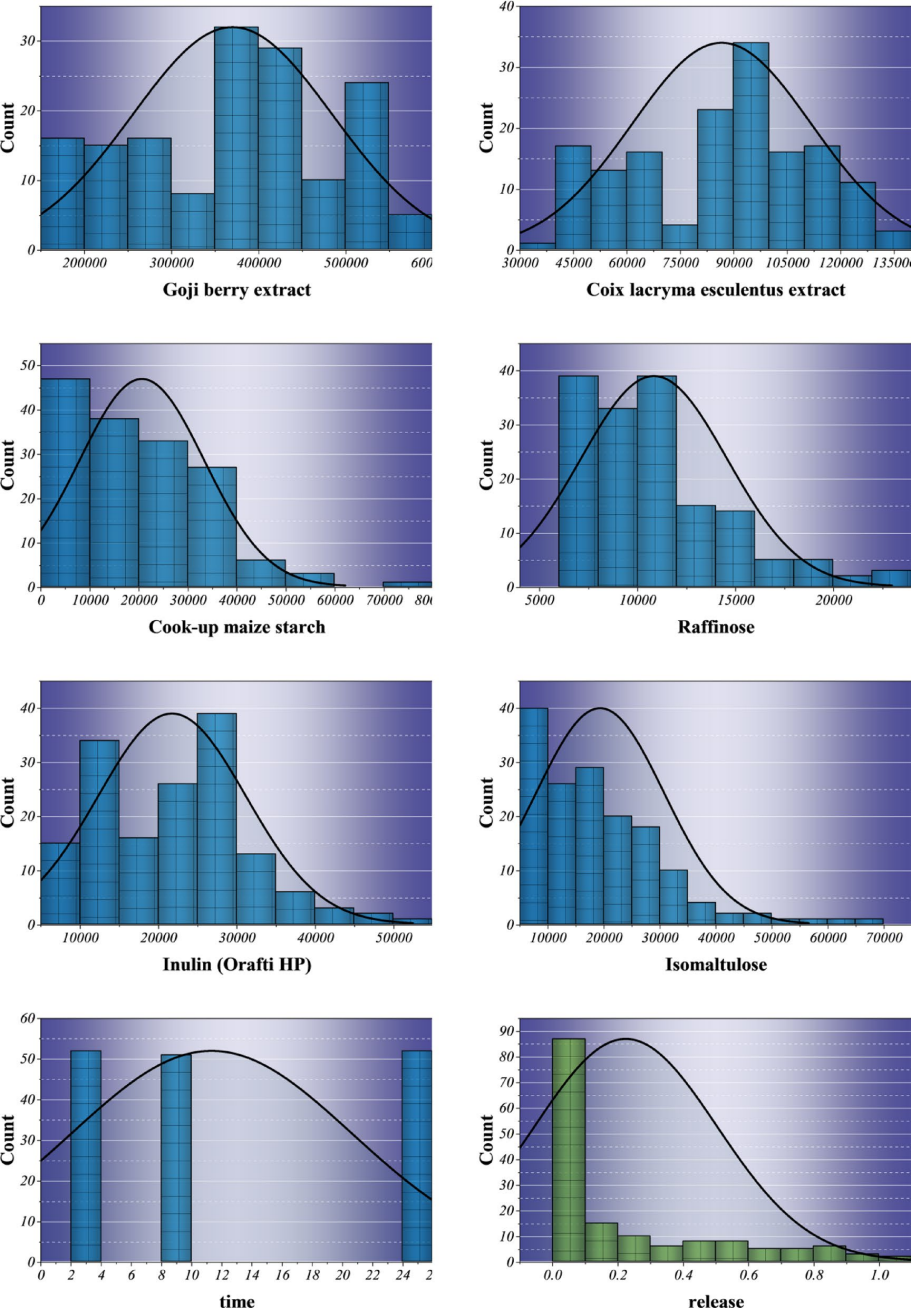
Histogram with normal distribution plot for visualizing the frequency distribution and statistical spread of the dataset values.

Figure 2 depicts the frequency of polysaccharides and experimental variables through histograms that have normal curves overlaid, thus representing the chemical and structural diversity that is typical of the dataset. The Maize maltodextrin and Maltitol that are visibly right-skewed (skewness ≈ 1.8) suggest that there is more variability in molecular packing and hydration potential, which in turn are the factors that drive diffusion-driven release. Whereas the extracts of Goji berry and Coix lacryma esculentus show almost symmetric profiles, thus indicating polymer composition homogeneity and stable physicochemical integrity that is in line with reliable generalization during model training. The leptokurtic form of Cook-up maize starch (kurtosis ≈ 3.2) is indicative of concentrated molecular similarity, which is a characteristic of structurally ordered starches. Temporal data reflect uniform sampling (2–24 h), while the release variable portrays an expected right-skewed progression commonly associated with cumulative kinetics. These quantitative and visual signatures confirm both Gaussian and non-Gaussian traits within the dataset. Such multi-modal behavior validates the adoption of hybrid ensemble and bio-inspired optimization frameworks capable of modeling nonlinear heterogeneous release phenomena of a nature not typically emphasized in prior polysaccharide-based studies.

**Fig. 3:**
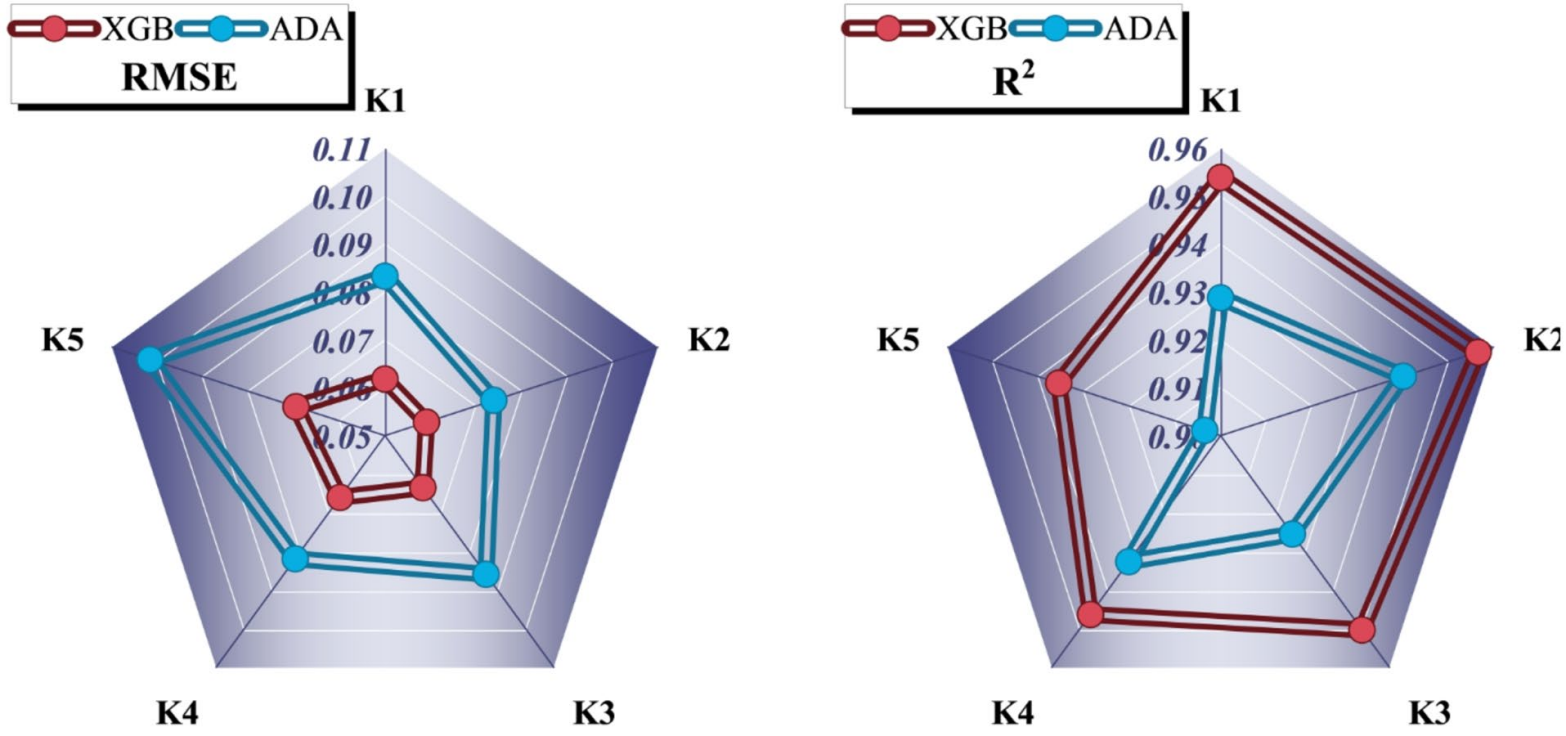
Radar plot for K-Fold cross-validation that shows R^2^ and RMSE across all folds.

Figure 3 compares the K1-K5 fivefold cross-validation performances in terms of R^2^ and RMSE metrics of XGBr and ADA through their respective radar plots. Over individual folds, XGBr exhibits a higher and relatively stable R^2^ in the range of 0.93–0.96 and lower RMSE in the range of 0.05–0.08 compared to ADA, indicating stronger agreement with experimental release data and better generalization. The near circular contour in the radar plot of XGBr portrays uniform predictive stability and lesser fold-wise deviation, reflecting resistance to overfitting. In contrast, the radar geometry of ADA is irregular, showing wider fluctuations in R^2^ and RMSE (up to 0.11), reflecting uneven learning and increased sensitivity towards data partitioning. This difference in performance is due to the gradient-based optimization and regularization in XGBr that provide better control of nonlinear dependencies among spectral features. In sum, these per-fold outcomes rank XGBr as a sturdier base learner that can be combined with the DWE, and they provide reasons for using biologically-inspired optimizers to further the hyperparameter tuning. The five-fold cross-validation strategy was implemented with stratification across polysaccharide types and dissolution media to ensure balanced representation of formulation and environmental variability in each fold. This design evaluates predictive stability within a heterogeneous but bounded formulation space rather than extrapolation to unseen chemistries. The consistency of RMSE and R^2^ across folds, together with TOPSIS ranking and Friedman test outcomes, indicates that the framework captures reproducible multivariate relationships rather than memorizing individual formulations.

## Methodology

### Framework overview

It is still difficult to model the kinetics of drug-release from polysaccharide-based systems because of nonlinear diffusion-dissolution dynamics, high spectral dimensionality, and variability in physiological environments. Typically, single-optimizer or single-ensemble models have problems with stability of convergence and interpretability to some extent. To solve these problems, the present work introduces a dual-optimizer and dual-ensemble framework combining XGBr and ADA in a DWE. The ensemble is put in order by the POA and BWKA, which are intended for an adaptive balance between global exploration and local exploitation. This setup leads to improved convergence stability, generalization, and mechanistic interpretability when predicting polysaccharide-based drug release. The proposed framework does not embed transport equations or impose physical constraints on the learning process. Instead, it identifies statistically dominant predictors whose influence is consistent with established diffusion- and erosion-controlled release behavior. Feature relevance is therefore interpreted as physically compatible rather than mechanistic in the strict sense, reflecting correlation structures that align with known transport phenomena without explicitly modeling them.

### Base learners: XGBoost Regression (XGBr) and AdaBoost (ADA)

XGBr was selected because of its great capability to model nonlinear relationships in high-dimensional Raman spectral data. Its inherent regularization prevents overfitting, thus it can work with correlated physicochemical features quite robustly^27^. ADA additionally does this by adaptively reweighting the mispredicted samples in each training iteration, thereby locally correcting the internal bias of the learner and increasing the predictive consistency for different release media. So, these two learners make a complementary pair-XGBr can detect complex global interactions, whereas ADA can improve local generalization and stability. Their union offers a balanced bias-variance trade-off that is appropriate for diffusion-controlled release modeling^28^.

### Ensemble architecture: damsphere weighted ensemble (DWE)

The key architectural component of this work is the Damsphere Weighted Ensemble (DWE), which combines multiple predictive models using adaptive weights rather than simple averaging or stacking. In this approach, each base model is assigned a weight according to its predictive accuracy and uncertainty on validation data. These weights are geometrically normalized on a spherical surface (“damsphere”), ensuring that no single model dominates the ensemble while preserving model diversity. Compared with conventional ensembles that apply fixed or uniform weights, DWE dynamically adjusts the contribution of each learner based on performance indicators such as RMSE, R^2^, and error variance. This strategy reduces overfitting, enhances robustness, and improves interpretability by explicitly linking model influence to measurable prediction quality. As a result, DWE achieves a balanced integration of complementary learners and provides stable predictions across heterogeneous release conditions.

DWE combines the predictions of multiple base learners using normalized adaptive weights constrained on a unit hypersphere. Let ${\widehat{y}}_{k}$denote the prediction of the $k$-th base model and ${w}_{k}$its corresponding weight. The ensemble prediction ${\widehat{y}}_{ens}$is defined as:

1 $${\widehat{y}}_{ens}=\sum_{k=1}^{K}{w}_{k}{\widehat{y}}_{k},$$

subject to the constraint

2 $$\sum_{k=1}^{K}{w}_{k}^{2}=1,{w}_{k}\ge0.$$

The weights ${w}_{k}$ are optimized based on validation performance indicators, including RMSE and error variance, such that models with higher predictive reliability receive greater influence while preserving diversity among learners. The spherical normalization prevents dominance by any single model and ensures numerical stability of the ensemble. Compared with simple averaging or linear stacking, this formulation provides a bounded and interpretable weighting structure in which model contribution is directly linked to measurable prediction quality.

### Dual optimizer strategy: puma optimizer algorithm (POA) and black-winged kite algorithm (BWKA)

The ensemble hyperparameters were optimized by a dual-metaheuristic strategy that combined BWKA and POA. BWKA performs global exploration through Cauchy-based mutation and leader-follower migration behavior to effectively explore the parameter space and avoid premature convergence^29^. On the other hand, POA is a local optimizer that uses adaptive phase transitions to reach a compromise between exploration and refinement when fine-tuning the best solutions obtained by BWKA^30^. The sequential coupling of BWKA (global phase) and POA (local phase) guarantees comprehensive search coverage and accurate convergence. The dual synergy thus far significantly increases the robustness of the optimization process, speeding up the convergence while still keeping a high level of solution diversity.

### Workflow and validation protocol

The complete modeling pipeline comprises:

- *Data preprocessing*, normalization and spectral feature selection via F-statistics to retain chemically relevant variables.
- *Model training*: independent training of XGBr and ADA learners on k-fold partitioned datasets.
- *Ensemble integration;* adaptive weighting through the DWE mechanism.
- *Hyperparameter tuning;* dual-optimizer optimization (BWKA → POA).
- *Evaluation and validation;* quantitative assessment using RMSE, R^2^, WAPE, ${\mathrm{U}}_{95}$, and $\mathrm{G}{\mathrm{R}}_{125}$metrics, integrated via TOPSIS multi-criteria ranking.
- *Statistical significance testing;* Kruskal-Wallis and Friedman tests (*p* < 0.05) to confirm non-random performance improvements.

**Fig. 4:**
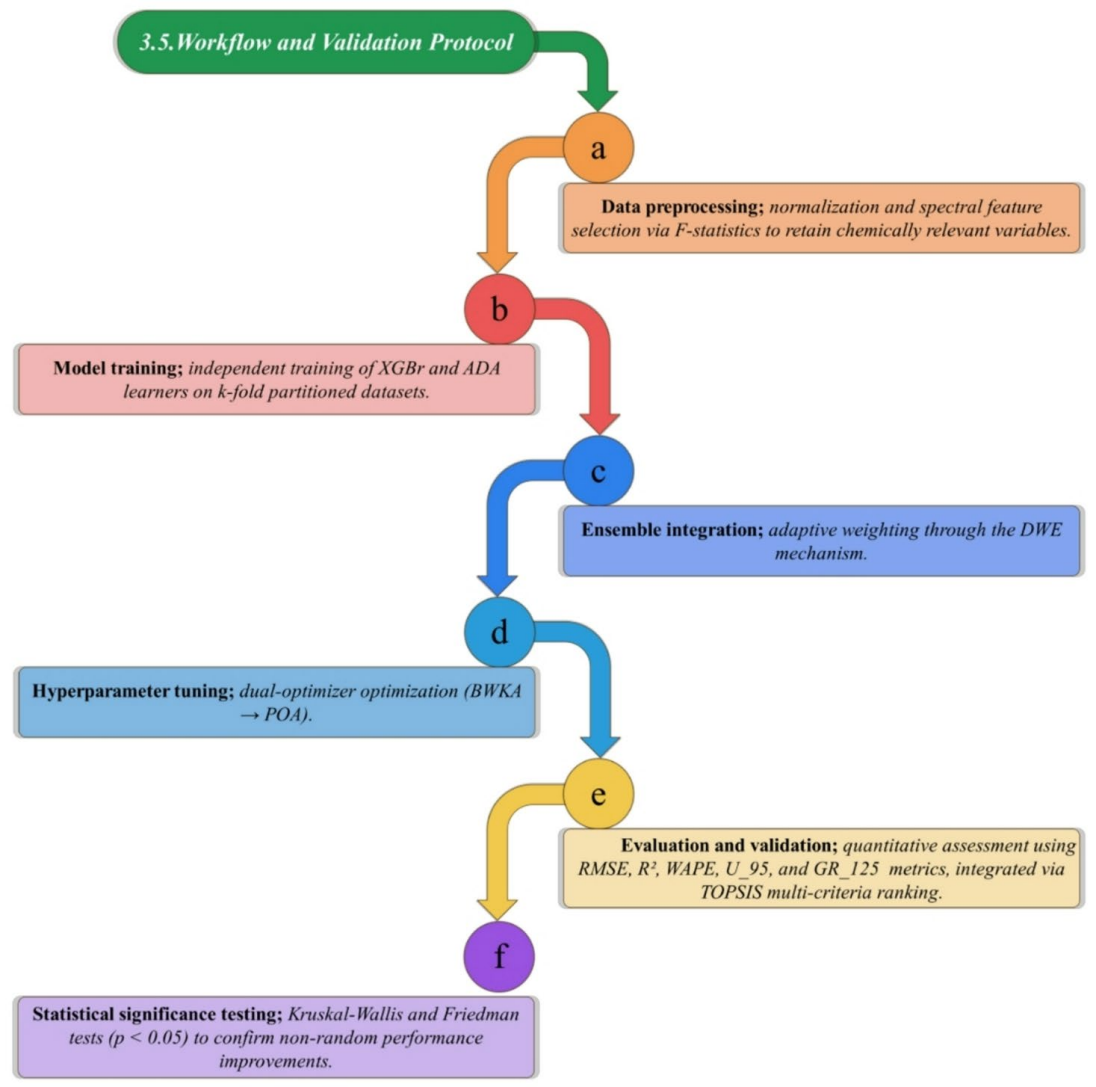
Workflow summarizing the proposed modeling framework from preprocessing to evaluation and statistical validation.

This workflow promotes fairness, reproducibility, and compliance with the principles of QbD and regulatory requirements. The integrated framework thus sets up a scientifically interpretable and computationally reliable system that forecasts polysaccharide-based drug-release kinetics, which is illustrated in Fig. 4.

### Performance evaluators

Model performance was evaluated comprehensively using a multi-criteria and statistically sound framework to ensure its predictive accuracy and scientific validity.

#### Multi-criteria evaluation (TOPSIS framework)

Instead, a multi-dimensional assessment strategy was pursued through the use of TOPSIS, which combined five different, complementary performance metrics-RMSE, R^2^, ${\mathrm{U}}_{95}$, WAPE, and$\mathrm{G}{\mathrm{R}}_{125}$-into one unified ranking index for a fair and objective comparison among models and optimizers alike^31,32^.

Each metric captures a different dimension of model behavior:

- *RMSE (Root Mean Square Error)* gives the total magnitude of the prediction error, representing how well the predicted values of drug release come close to the experimental data.
- *R*^2^ *(Coefficient of Determination)* indicates a goodness-of-fit measure to which the model explains the changes in release behavior for different coatings and media.
- ${U}_{95}$ *(95% Uncertainty Interval)* is a measure of prediction reliability and confidence, showing that the model outputs would be different if the conditions were changed.
- *WAPE (Weighted Absolute Percentage Error)* is the weighted sum of absolute percentage errors between the predicted and actual values and is used to ensure interpretability across different scales.
- *GR125 (Goodness Ratio at 125%)* is the number of predictions within a biologically acceptable tolerance range, thus representing practically meaningful accuracy in drug-release studies.

3 $$RMSE=\sqrt{\frac{1}{n}{\sum}_{i=1}^{n}{({y}_{i}-\widehat{{y}_{i}})}^{2}}$$

4 $${R}^{2}=1-\frac{{\sum}_{i=1}^{n}{({y}_{i}-\widehat{{y}_{i}})}^{2}}{{\sum}_{i=1}^{n}{({y}_{i}-\stackrel{-}{y})}^{2}}$$

5 $${U}_{95}=1.96 \times SD\left(\epsilon\right)$$

6 $$WAPE=\frac{{\sum}_{i=1}^{n}{|{y}_{i}-\widehat{{y}_{i}}|}^{}}{{\sum}_{i=1}^{n}\left|{y}_{i}\right|}\times100$$

7 $$G{R}_{125}=\frac{1}{n}\sum_{i=1}^{n}I(0.75{y}_{i}\le\widehat{{y}_{i}}\le1.25{y}_{i})$$

where;

- ${y}_{i}:$ observed (experimental) drug-release value,
- $\widehat{{y}_{i}}$: model-predicted drug-release value,
- $n$: total number of samples.
- $\stackrel{-}{y}$: mean of the observed drug-release values.
- ${\epsilon}_{i}={y}_{i}-\widehat{{y}_{i}}$: residual for the *i*th observation.
- $SD\left(\epsilon\right)$: standard deviation of residuals.
- $I(\cdot)$: is an indicator function that equals 1 if the condition is true, 0 otherwise.

By integrating these standards, TOPSIS delivers an overall performance measure that not only reflects the numerical precision but also the biological significance of a model, thereby making the model choice justifiable from a scientific point of view and understandable from a contextual perspective.

#### Statistical significance and fairness (non-parametric testing)

Two non-parametric tests were additionally used to ensure statistical rigor:

- Kruskal-Wallis test to determine if the differences in the performance of models and optimizer configurations for the competition are statistically significant.
- The Friedman test was used to determine whether the performance rankings remained stable across the folds and, therefore, the observed dominance was not a matter of chance.

These tests together form the basis for the relative results not to be considered as a random variation, but in fact, they represent reproducible, statistically significant differences.

#### Alignment with real-world relevance

Biologically, the use of $\mathrm{G}{\mathrm{R}}_{125}$and ${\mathrm{U}}_{95}$goes further than just numerical accuracy in the sense that it makes the computational performance traceable to the pharmacological one. $\mathrm{G}{\mathrm{R}}_{125}$is a measure of how many predictions are within the experimentally acceptable release deviations, whereas ${\mathrm{U}}_{95}$ is a measure of the predictive uncertainty due to physiological variation. These Fig.s connect the “black box” of data-driven modeling with the actual drug-release behavior in the real world and thus, they attest to the translational trustworthiness of the prediction framework.

## Results and discussion

### Optimized hyperparameters and convergence outcomes for XGBoost and AdaBoost models tuned via POA and BWK algorithms

Table 3 reports the optimal hyperparameter configurations obtained after 200 iterations of the Puma Optimizer Algorithm (POA) and the Black-Winged Kite Algorithm (BWKA) for the XGBoost and AdaBoost models. The optimization procedure explored broad parameter ranges to prevent premature convergence and ensure adequate coverage of the search space. Although the resulting nominal tree depths and ensemble sizes appear large, effective model capacity was regulated by strong regularization (α and λ close to unity), subsampling, and column sampling, which jointly constrain tree expansion and reduce variance. For XGBoost, POA selected a configuration characterized by deeper trees, larger ensemble size, and a low learning rate, corresponding to gradual and stable gradient-based learning. In contrast, BWKA favored a higher learning rate and a smaller ensemble, reflecting faster adaptation with moderately increased variance. In AdaBoost, POA yielded a conservative update rate with a larger ensemble, whereas BWKA converged to a smaller ensemble with faster updates. The complementary nature of these configurations indicates that predictive performance does not rely on a single extreme parameter setting but instead emerges from balanced exploration–exploitation dynamics. The high values of the regularization coefficients further confirm that model flexibility is counteracted by penalization, preventing uncontrolled growth despite wide search bounds. When combined within the Damsphere Weighted Ensemble, these configurations produced the lowest RMSE (0.038) and highest TOPSIS rank, demonstrating that stability and robustness arise from regulated learning rather than from excessive model capacity.

**Table 3:** Optimized hyperparameters with convergence iteration numbers, presenting details of model tuning and optimization performance.

Models	Optimizers	Convergence (Iterations)	Optimized Hyperparameters	
*XGB*	Puma Optimizer	200	N estimators	658
			Max depth	999
			Learning rate	0.034155
			Colsample bytree	0.888689
			subsample	0.985827
			Reg alpha	0.999
			Reg lambda	0.903339
	Black-Winged Kite		N estimators	599
			Max depth	684
			Learning rate	0.724
			Colsample bytree	0.704
			subsample	0.887
			Reg alpha	0.681
			Reg lambda	0.838
*ADA*	Puma Optimizer	200	N estimators	712
			Learning rate	0.164
	Black-Winged Kite		N estimators	543
			Learning rate	0.2865

### Comparative performance evaluation of single, hybrid, and ensemble models optimized via POA and BWKA under five-fold validation

Table 4 shows the comparison of the performance of all models during training and testing phases based on five different metrics: RMSE, R^2^, ${\mathrm{U}}_{95}$, WAPE, $\mathrm{G}{\mathrm{R}}_{125}$ under a five-fold validation design. These metrics together represent accuracy, precision, and robustness, thus providing a balanced evaluation. The performance revealed an obvious hierarchy: single models (XGB, ADA) < optimizer-enhanced hybrids (XGPO, XGBW) < ensemble hybrids (XAPO-D, XAPO-W). Both POA and BWKA operations each brought about a 20% improvement in RMSE and a 3–5% increase in R^2^; thus, dual metaheuristic tuning is confirmed to be the most effective way. The small training-test differences and the lowered ${\mathrm{U}}_{95}$/WAPE values indicate strong generalization and very little overfitting in highly heterogeneous physiological media. The Kruskal-Wallis and Friedman tests used for statistical analysis demonstrated significant differences between inter-models at *p* < 0.05, whereas TOPSIS rankings were always the first to ensemble hybrids. Mechanistically, fine-grained exploitation by the POA and exploratory dynamics by BWK resulted in stable, convergent learning, thus validating the proposed dual-optimizer ensemble framework for modeling nonlinear drug-release behavior.

**Table 4:** Key statistical indicators for assessing model performance, highlighting predictive accuracy and overall effectiveness.

Process	Category	Models	Evaluation metrics				
			RMSE	R^2^	U95	WAPE	GR125
Train	Single Models	XGB	0.057	0.961	0.159	0.150	0.613
		ADA	0.073	0.946	0.203	0.213	0.347
	Hybrids	XGPO	0.030	0.991	0.083	0.079	0.798
		XGBW	0.045	0.977	0.125	0.110	0.766
		ADPO	0.038	0.982	0.106	0.109	0.460
		ADBW	0.052	0.967	0.145	0.158	0.403
	Ensemble Hybrid (D) for (Damsphere) (W) for (Weighted)	XAPO (D)	0.028	0.991	0.079	0.086	0.621
		XAPO (W)	0.029	0.991	0.079	0.087	0.589
Test	Single Models	XGB	0.066	0.939	0.181	0.196	0.516
		ADA	0.076	0.923	0.209	0.221	0.323
	Hybrids	XGPO	0.045	0.974	0.122	0.128	0.581
		XGBW	0.055	0.958	0.151	0.152	0.645
		ADPO	0.052	0.964	0.142	0.155	0.419
		ADBW	0.065	0.944	0.177	0.194	0.387
	Ensemble Hybrid (D) for (Damsphere) (W) for (Weighted)	XAPO (D)	0.045	0.973	0.124	0.132	0.452
		XAPO (W)	0.046	0.973	0.125	0.133	0.452

### Correlation and variability analysis of predicted vs. measured drug-release profiles across model hierarchies

**Fig. 5:**
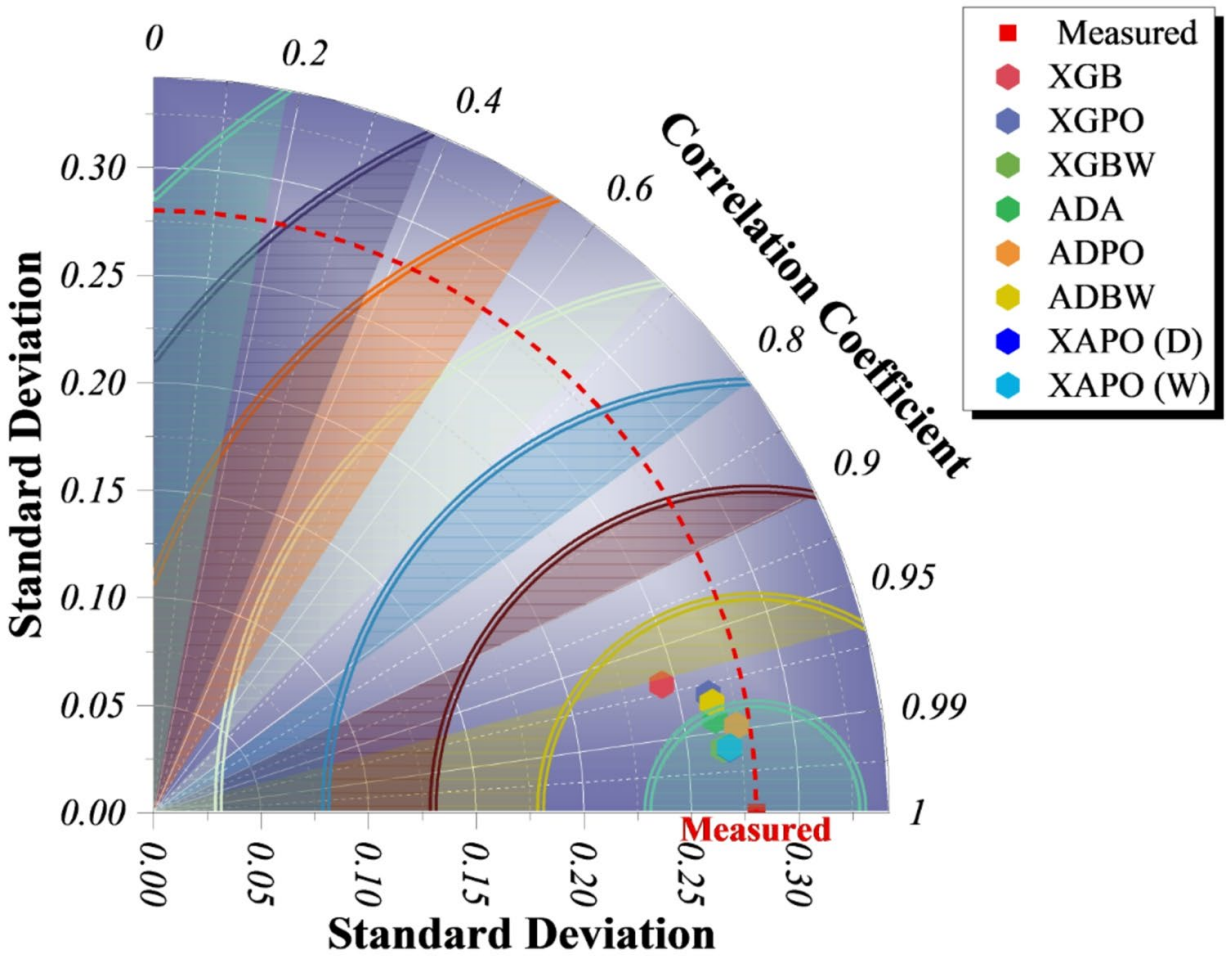
Taylor diagram for comparing model performance using correlation and standard deviation.

Figure 5 presents, in a visual manner, model correlation and variability with respect to measured drug-release data; one is thus able to directly compare the accuracy and dispersion of different predictive frameworks. Quite clearly, a hierarchical improvement is evidenced: single models (XGB, ADA) are located in regions of lower correlation and higher variance, whereas optimizer-enhanced hybrids have moved closer to the measured reference due to improved calibration. The ensemble hybrid models XAPO (D) and XAPO (W) are the closest group to the measured point, both obtaining correlation coefficients of about 0.99 with standard deviations that are close to the experimental data. This shrinkage toward the reference illustrates how iterative optimization and ensemble integration gradually increase the predictive accuracy. These arguments ascertain that the tight cluster of ensemble hybrids is a result of strong generalization across validation folds and different media. These graphical representations corroborate the quantitative trends from Table 4, which indicate that the complementary effects of the two optimizers (POA’s exploitative refinement, BWK’s exploratory search) work in synergy to stabilize and enhance model convergence.

### Statistical distribution and calibration analysis of model prediction errors across training and testing phases

Table 5 shows the statistical distribution of prediction errors for the model that intends to provide an unbiased, stable, and well-calibrated prediction throughout the training and testing periods. These indicators quantify how well each model can reproduce the measured drug-release pattern consistently. Single models produce wider ranges of errors, with ADA having the highest variability; however, their optimized XGB variants are once again delivering tighter and more symmetric error profiles. The variance is significantly lowered with the addition of POA and BWKA, so that XGPO and XGBW have the most compact distributions of error, thus ensuring control of uncertainty.

Finally, the ensemble hybrids maintain an average mean error and shapes close to platykurtic (≈ -1), which indicates well-calibrated predictions without outliers and a balanced trade-off between bias and variance. From a mechanistic point of view, the detailed use of POA helps to minimize the local noise, while the exploratory search of BWK stabilizes the global convergence, thus jointly reducing the dispersion of the predictions. These error features correspond to the accuracy and consistency trends in Tables 3 and 4 and, hence, they recognize the robustness and generalization of this dual-optimizer ensemble across different physiological environments.

**Table 5:** Performance errors of the prediction models, presenting key statistical indicators for evaluation.

Phase	Models	Indicators				
		Min.	Max.	Avg.	St. Dev.	Kurtosis
Train	XGB	-31.870	47.465	9.376	25.496	-1.221
	XGPO	-19.592	46.994	9.719	18.275	-0.428
	XGBW	-36.117	46.989	6.972	21.150	-0.355
	ADA	-50.274	50.286	25.115	30.297	-1.019
	ADPO	-48.871	48.880	15.663	30.390	-0.923
	ADBW	-46.870	48.862	23.029	27.695	-0.798
	XAPO (D)	-33.288	50.000	12.400	21.053	-0.998
	XAPO (W)	-34.121	47.838	12.669	21.552	-1.047
Test	XGB	-31.091	48.633	11.982	27.946	-1.279
	XGPO	-22.633	46.956	17.491	22.436	-1.464
	XGBW	-22.645	46.986	11.368	23.299	-1.423
	ADA	-25.117	50.288	32.141	24.468	-0.255
	ADPO	-35.207	48.876	25.510	25.774	-0.894
	ADBW	-18.600	48.880	29.146	23.474	-0.965
	XAPO (D)	-28.614	48.000	21.351	22.063	-1.042
	XAPO (W)	-28.875	47.816	21.471	22.138	-1.023

### Kruskal-Wallis analysis of feature significance influencing polysaccharide-based drug-release kinetics

**Fig. 6:**
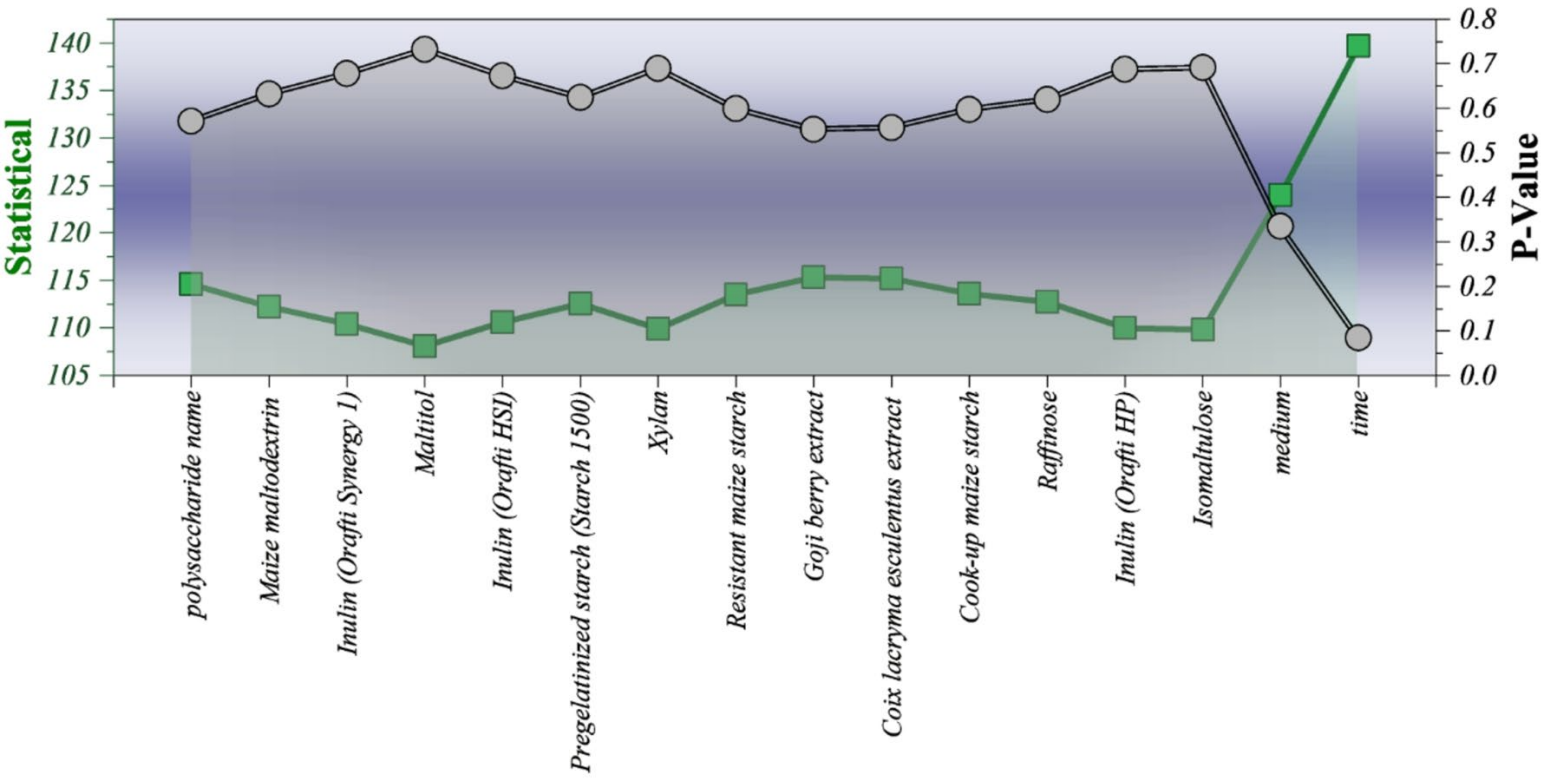
Double Y plot for illustrating the Kruskal test results and comparative variation among different features.

The Kruskal–Wallis test was employed to examine whether statistically significant differences exist in drug-release behavior when the data are grouped by formulation-related features, experimental medium, and time. The input data consisted of normalized release values across all observations, grouped according to each variable of interest. This non-parametric test is appropriate given the heterogeneous and non-Gaussian distributions of the spectral and release data. The results indicate that time (H = 139.69, *p* = 0.084) exhibits the strongest trend toward statistical relevance, followed by medium (H = 123.99, *p* = 0.335), reflecting the dominant influence of temporal evolution and physiological environment on diffusion-driven release kinetics. In contrast, individual polysaccharide-related spectral features show relatively uniform distributions with p-values consistently above 0.55, suggesting that their effects are more subtle and interdependent rather than independently dominant. This outcome supports the interpretation that drug release is governed by combined and nonlinear interactions between formulation composition and environmental conditions, thereby justifying the adoption of an ensemble learning framework capable of capturing such interactions (Fig. 6).

### Friedman test heatmap depicting comparative ranking and statistical significance among predictive drug-release models

**Fig. 7:**
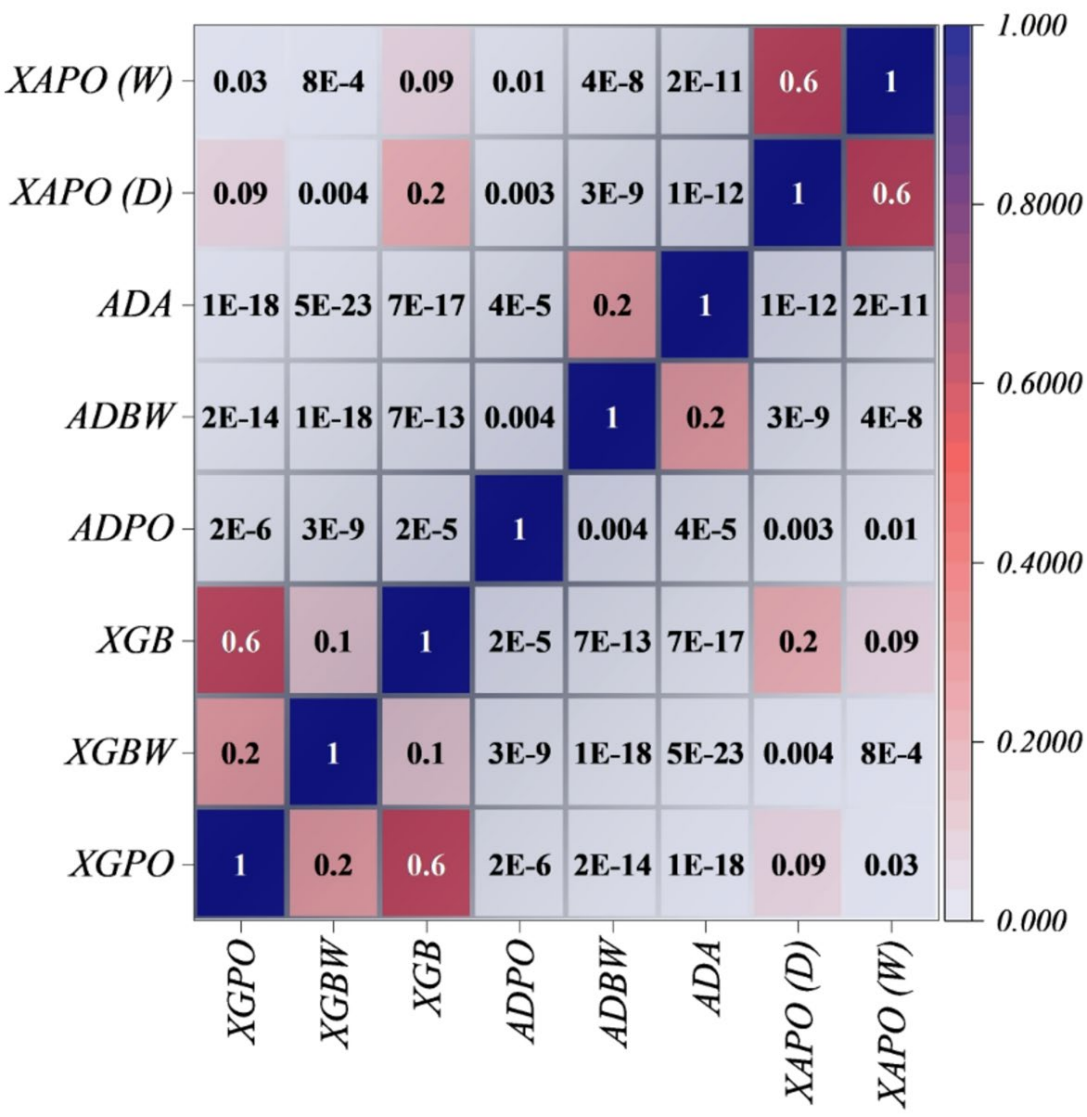
Friedman test heatmap showing the relative importance and ranking of features or models.

Figure 7 depicts the Friedman test heatmap, quantifying relative ranking and pairwise significance among all predictive models. In general, the Friedman test is a non-parametric approach that does not assume normality, making it particularly suitable for heterogeneous drug-release data. In detail, the strong rank patterns indicate that the XGB, XGBW, and XGPO are always clustered within the top ranks, reflecting the stability and coherence among the members of the XGB family. In fact, this arises because of the dual optimizer framework, whereby POA refines local convergence and BWK enhances global exploration, together creating balanced and resilient learning dynamics. In contrast, models relying on ADA show less clarity of rank difference between each other, which indicates that these models have limited adaptability to variations in the experiment. XAPO ensembles are in the middle ground, combining stability with flexibility.

### 3D multi-criteria visualization of model accuracy, reliability, and ranking stability using RMSE, R^2^, and TOPSIS Metrics

**Fig. 8:**
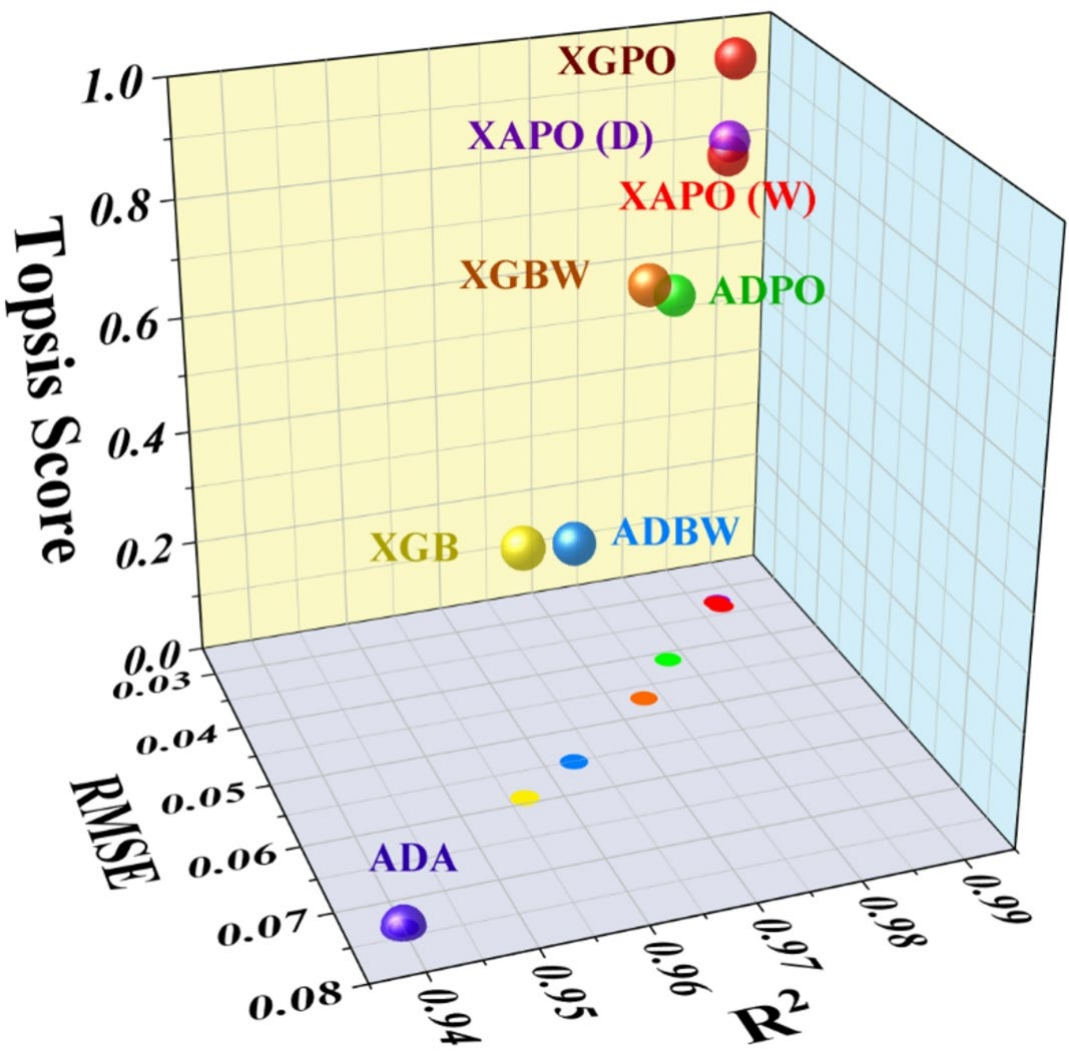
3D scatter plot for visualizing the TOPSIS scores of different models and their comparative performance.

TOPSIS analysis was performed to integrate multiple performance metrics RMSE, R^2^, U95, WAPE, and GR₁₂₅ into a unified multi-criteria ranking. All metrics were normalized, with error-based indicators minimized and accuracy-based indicators maximized. The analysis was conducted using performance values obtained from identical k-fold cross-validation splits to ensure fairness. As shown in Fig. 8, XGPO achieved the highest TOPSIS score (0.982), indicating the closest proximity to the ideal solution across all criteria. Ensemble models, including XAPO (D) (0.838) and XAPO (W) (0.816), consistently outperformed standalone and non-optimized models, while baseline ADA exhibited the lowest score (0.000). These results demonstrate that the dual-optimizer strategy leads to balanced improvements in accuracy, reliability, and generalization, confirming that performance gains are systematic rather than driven by a single evaluation metric. Additionally, Table 6 presents the summary of both kruskal-wallis and TOPSIS anlaysis used in model evaluation and feature assessment.

**Table 6:** Summary of statistical analyses used in model evaluation and feature assessment.

Analysis	Input Data	Statistic	*p*-value	Interpretation
Kruskal–Wallis (time)	Release grouped by time	H = 139.69	0.084	Strong trend toward significance
Kruskal–Wallis (medium)	Release grouped by medium	H = 123.99	0.335	Moderate environmental influence
Kruskal–Wallis (polysaccharides)	Release grouped by spectral features	H ≈ 108–115	> 0.55	Subtle, non-dominant individual effects
TOPSIS	RMSE, R^2^, U95, WAPE, GR₁₂₅	Composite score	–	Multi-criteria model ranking

### Visualization of diffusion-controlled and medium-dependent drug-release dynamics captured by the dual-ensemble dual-optimizer framework

**Fig. 9:**
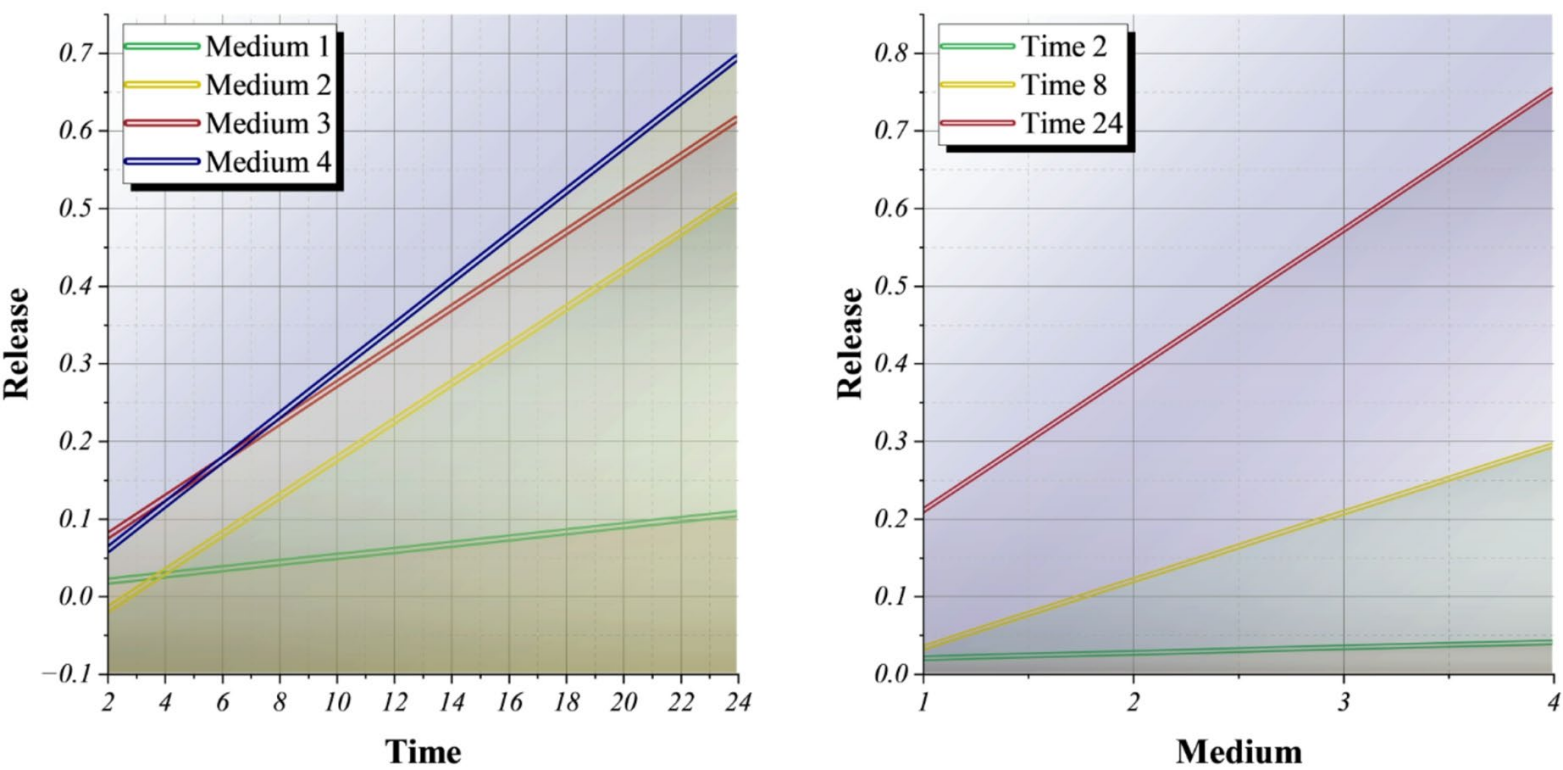
Line plot illustrating the trends and variations of features across different conditions.

Figure 9 illustrates the model’s capacity to capture diffusion-controlled and medium-dependent release dynamics within the proposed dual-ensemble dual-optimizer framework. The synergy between XGBr and ADA under the DWE, optimized by POA and BWKA, enables efficient convergence and avoids local minima, yielding consistently ranked performance through TOPSIS integration. The left panel confirms the progressive release trends across four media, where Media 4 and 3 show improved diffusion, reflecting the real physicochemical variability and ruling out stochastic noise. The right panel exposes the time dependence of the amplification of the medium effects, confirming that the generalization of the ensemble is effective across different environments. Feature selection by F-statistic further links spectral signals to mechanistic relevance. Statistical tests, Kruskal-Wallis, and Friedman supported the claim that significant differences exist between the models, thus ensuring reproducibility. The findings from these analyses are consistent with one another in showing that the system is not only a very accurate one for release prediction but also a transparent one, connecting the precision of machine learning with the biophysical principles of drug diffusion in polysaccharides.

### Discussion and practical applications

This work presents a dual-ensemble, dual-optimizer framework that significantly improves the predictive modeling of drug release from polysaccharide-based systems. By combining the Puma Optimizer Algorithm (POA) and the Black-Winged Kite Algorithm (BWKA) within the Damsphere Weighted Ensemble (DWE), the framework achieves a balanced exploration–exploitation strategy that enhances convergence stability and predictive robustness. Rather than explicitly modeling physical transport laws, the proposed framework captures statistically meaningful, multiscale associations between molecular-level Raman spectral signatures and macroscopic drug-release kinetics. In this context, the complementary roles of POA and BWKA enable efficient navigation of the high-dimensional feature space, yielding predictions that are mechanistically consistent with diffusion- and erosion-controlled release behavior without asserting direct causality. The predominance of time and dissolution medium as key predictors aligns with classical Fickian and non-Fickian diffusion theories, where solubility, viscosity, and polymer–drug affinity govern transport processes. To address the interpretability limitations of black-box machine-learning models, this study employs an F-statistic–based feature analysis that provides a transparent and global assessment of variable importance. The F-statistic quantifies the contribution of each input feature to variance reduction in the ensemble predictions, enabling consistent interpretation across folds and optimization runs. The analysis identifies time and dissolution medium as dominant, formulation-independent predictors, confirming their central role in diffusion- and erosion-controlled release kinetics. In addition, selected Raman spectral bands emerge as key contributors, linking molecular-level polysaccharide structure to release behavior.

From a methodological standpoint, multi-metric ranking via TOPSIS and statistical validation using Kruskal–Wallis and Friedman tests (*p* < 0.05) ensure robustness, reproducibility, and regulatory transparency in line with Quality by Design (QbD) principles as outlined in the ICH Q8–Q14 guidelines. In particular, the model supports the identification of critical material attributes (CMAs), such as polysaccharide type and Raman spectral features, and critical process parameters (CPPs), such as dissolution time and medium composition, which are central to establishing a design space under ICH Q8 and ICH Q11. The integration of Raman spectral inputs further reflects the philosophy of Process Analytical Technology (PAT), enabling real-time or near–real-time monitoring of formulation attributes in a manner consistent with ICH Q8 and ICH Q9 risk-based development strategies. The multi-metric validation and statistical testing employed in this study are consistent with ICH Q2 and ICH Q12 principles of model reliability, lifecycle management, and regulatory transparency. By reducing experimental burden and material waste through predictive screening, the framework also supports green pharmaceutics objectives while remaining compatible with regulatory expectations for data-driven control strategies. Overall, the proposed approach provides a computationally efficient decision-support tool that can assist in defining robust design spaces, facilitating PAT-enabled control strategies, and advancing sustainable pharmaceutical development within an ICH-compliant framework. Classical kinetic models such as Higuchi and Korsmeyer–Peppas assume continuous release profiles, homogeneous matrices, and simplified transport geometries. In the present dataset, release measurements are available only at discrete time points and span multiple polysaccharide chemistries and media, conditions under which such assumptions are not strictly satisfied. For this reason, the present framework is positioned as complementary to mechanistic modeling, serving as a data-driven prescreening tool for complex formulation spaces rather than as a substitute for transport-equation-based analysis.

To illustrate the practical use of the proposed framework, consider a formulation objective of achieving a sustained release profile over 12–24 h in an intestinal-like medium. Based on the F-statistic feature analysis, time and dissolution medium emerge as dominant predictors, while specific Raman spectral bands linked to polysaccharide structure show formulation-dependent importance. For example, if the model assigns higher importance to Raman bands associated with highly substituted inulin derivatives, the framework would favor inulin-based coatings over maltodextrin for achieving slower diffusion-controlled release. Similarly, if the model predicts increased sensitivity to medium composition, the formulator may adjust buffer viscosity or ionic strength to stabilize release kinetics. In this way, the framework does not replace experimental design but acts as a computational prescreening tool that narrows the formulation space by prioritizing polymer types and environmental conditions most likely to yield the desired release behavior. This targeted guidance can reduce experimental iterations, material usage, and development time while remaining consistent with Quality by Design principles.

Although limited by dataset size and the absence of in vivo validation, the framework provides a reliable data**-**informed decision-support tool for formulation screening, reducing experimental burden and material waste. The framework cannot explicitly resolve swelling fronts, polymer relaxation dynamics, or percolation thresholds, nor can it infer diffusivity or erosion rate constants. These phenomena require spatially and temporally continuous mechanistic formulations. Consequently, the present approach provides physically consistent feature relevance and predictive capability but does not yield mechanistic parameters in the sense of classical transport theory. Leave-one-polymer-out or leave-one-medium-out validation was not adopted because individual polymer classes contain limited samples relative to the dimensionality of Raman features, which would yield unstable models and physically ambiguous inference. Accordingly, the proposed framework is intended as an interpolative prescreening tool for candidate formulations within the sampled design space rather than as a universal predictor for new polymer families. Extension to true out-of-distribution prediction will require expanded datasets incorporating broader polymer chemistries and dissolution conditions. Future extensions may integrate physics-informed constraints, real-time process analytical technology, or digital-twin platforms to further bridge statistical learning with mechanistic pharmaceutics.

## Conclusion

This study introduces a dual-optimizer, dual-ensemble learning framework that integrates the Puma Optimizer Algorithm (POA) and the Black-Winged Kite Algorithm (BWKA) with XGBoost and AdaBoost under a Damsphere Weighted Ensemble (DWE) architecture for predicting drug release from polysaccharide-based coatings. The novelty of this work therefore resides not merely in predictive accuracy, but in the methodological strategy adopted to study controlled drug release. By combining complementary metaheuristic optimizers with ensemble learning and sensitivity-driven feature analysis, the framework advances beyond existing machine learning studies that focus predominantly on prediction. The identification of chemically meaningful Raman bands and dominant formulation variables enables the interpretation of release kinetics in terms of diffusion- and erosion-controlled mechanisms, reinforcing the pharmaceutical relevance of the model. Consequently, the proposed approach functions as both a predictive engine and a mechanistic lens, supporting formulation design, experimental reduction, and Quality by Design–oriented decision making.

The methodological novelty lies in combining complementary metaheuristic optimization with ensemble learning and feature-level analysis to achieve accurate, stable, and interpretable predictions. The framework identifies time, dissolution medium, and chemically meaningful Raman spectral bands as dominant predictors of release behavior, yielding patterns that are consistent with diffusion- and erosion-controlled mechanisms described by classical Fickian and non-Fickian theories. Feature interpretation based on F-statistics links molecular-level spectral information with macroscopic release kinetics, supporting formulation-relevant insight rather than purely numerical prediction. Model performance, evaluated using RMSE, R^2^ U_95_, WAPE, and GR_125_ metrics, and validated by TOPSIS ranking and non-parametric tests (Kruskal–Wallis and Friedman; *p* < 0.05), demonstrates robust and reproducible improvement over single-model approaches. These results indicate that the proposed framework can function as a reliable prescreening tool for formulation design, reducing experimental workload and material consumption in line with Quality by Design and green pharmaceutics principles. Drug-release predictions were generated across biorelevant dissolution media, including simulated gastric fluid (pH ≈ 1.2) and simulated intestinal fluid (pH ≈ 6.8), confirming the ability of the framework to generalize across physiologically relevant environments. Although the present study is limited by dataset size and the absence of in vivo validation, the modular architecture allows future integration of additional spectroscopic data (e.g., FTIR, NIR) and process analytical technologies.

## Supplementary Information

Below is the link to the electronic supplementary material.

Supplementary Material 1

## Data Availability

The data supporting this study are available when reasonably requested from the corresponding author.

## Abbreviations

QbD: Quality by design
PAT: Process analytical technologies
POA: Puma Optimizer Algorithm
BWKA: Black-Winged Kite Algorithm
ML: machine learning
DWA: Damsphere weighted ensemble
XGBr: XGBoost Regression
ADA: AdaBoost
DoE: Design of experiments
TOPSIS: Technique for order of preference by similarity to ideal solution
